# Combination of Histone Deacetylase Inhibitor Panobinostat (LBH589) with β-Catenin Inhibitor Tegavivint (BC2059) Exerts Significant Anti-Myeloma Activity Both In Vitro and In Vivo

**DOI:** 10.3390/cancers14030840

**Published:** 2022-02-08

**Authors:** Ioanna Savvidou, Tiffany Khong, Sophie Whish, Irena Carmichael, Tara Sepehrizadeh, Sridurga Mithraprabhu, Stephen K. Horrigan, Michael de Veer, Andrew Spencer

**Affiliations:** 1 Myeloma Research Group, Australian Centre for Blood Diseases, Central Clinical School, Faculty of Medicine, Nursing and Health Sciences, Monash University, Melbourne, VIC 3004, Australia; tiffany.khong@monash.edu (T.K.); swhish88@gmail.com (S.W.); durga.mithraprabhu@monash.edu (S.M.); 2 Malignant Haematology and Stem Cell Transplantation, Department of Haematology, Alfred Hospital, Melbourne, VIC 3004, Australia; 3 Monash Micro Imaging-Alfred Research Alliance, Faculty of Medicine, Nursing and Health Sciences, Monash University, Melbourne, VIC 3004, Australia; iska.carmichael@monash.edu; 4 Monash Biomedical Imaging, Monash Technology Research Platforms, Monash University, Melbourne, VIC 3004, Australia; tara.sepehrizadeh@monash.edu (T.S.); michael.deveer@monash.edu (M.d.V.); 5 Iterion Therapeutics, 2450 Holcombe Blvd, Houston, TX 77021, USA; stephen@iteriontx.com; 6 Department of Clinical Hematology, Monash University, Melbourne, VIC 3004, Australia

**Keywords:** multiple myeloma, drug resistance, histone deacetylase inhibition, β-catenin inhibition, combination treatment, in vivo drug synergism

## Abstract

**Simple Summary:**

Multiple myeloma remains an incurable malignancy with the majority of patients succumbing to the disease after receiving several lines of treatment, while acquiring drug resistance. Although panobinostat, a histone deacetylase inhibitor, has long been approved for the treatment of patients with relapsed/refractory myeloma it has not yet been incorporated into everyday practice. In this study we showed a significant synergistic anti-myeloma effect of low dose panobinostat and β-catenin inhibitor Tegavivint both in vitro and in vivo, with a favourable toxicity profile. This combination could significantly and safely benefit myeloma patients that have exhausted mainstream therapeutic modalities due to acquisition of drug resistance in the future.

**Abstract:**

Over the last three decades changes in the treatment paradigm for newly diagnosed multiple myeloma (MM) have led to a significant increase in overall survival. Despite this, the majority of patients relapse after one or more lines of treatment while acquiring resistance to available therapies. Panobinostat, a pan-histone deacetylase inhibitor, was approved by the FDA in 2015 for patients with relapsed MM but how to incorporate panobinostat most effectively into everyday practice remains unclear. Dysregulation of the Wnt canonical pathway, and its key mediator β-catenin, has been shown to be important for the evolution of MM and the acquisition of drug resistance, making it a potentially attractive therapeutic target. Despite concerns regarding the safety of Wnt pathway inhibitors, we have recently shown that the β-catenin inhibitor Tegavivint is deliverable and effective in in vivo models of MM. In this study we show that the combination of low concentrations of panobinostat and Tegavivint have significant in vitro and in vivo anti-MM effects including in the context of proteasome inhibitor resistance, by targeting both aerobic glycolysis and mitochondrial respiration and the down-regulation of down-stream β-catenin targets including myc, cyclinD1, and cyclinD2. The significant anti-MM effect of this novel combination warrants further evaluation for the treatment of MM patients with relapsed and/or refractory MM.

## 1. Introduction

Multiple Myeloma (MM) is the second most common haematologic malignancy with 176,404 new cases and 117,077 deaths registered worldwide in 2020 [1]. It arises from the malignant transformation of antibody producing plasma cells residing in the bone marrow and is preceded by the premalignant condition MGUS (Monoclonal Gammopathy of Undetermined Significance). While the treatment landscape for MM has changed significantly over the past 30 years with the introduction of proteasome inhibitors (bortezomib, carfilzomib, ixazomib), immunomodulatory drugs (IMiDs) (thalidomide, lenalidomide, pomalidomide) and monoclonal antibodies (daratumumab and elotuzumab), resulting in improved overall survival (OS) [2] most patients eventually relapse, with the duration and depth of response decreasing with each line of therapy [3].

Panobinostat (LBH589) is an oral pan-histone deacetylase (HDAC) inhibitor that, based on the results of the PANORAMA 1 trial [4], was approved by the FDA in 2015 for the treatment of patients with relapsed and/or refractory MM (RRMM) who had received two or more prior lines of therapy, including bortezomib and an IMiD. More recently, the results of the PANORAMA 3 trial exploring alternative dosing and scheduling of panobinostat in combination with sub-cutaneous bortezomib have confirmed both a high level of efficacy and the deliverability of this combination in RRMM [5]. This confirmation provides a rationale for the further evaluation of panobinostat as a novel therapeutic modality in treatment combinations with alternative mechanisms of action to address the ongoing unmet need for more effective therapies for RRMM [6].

The role in tumorigenesis of dysregulation of canonical Wnt signalling and its key player β-catenin have long been recognized [7]. In relapsed MM, studies have confirmed aberrant activation of the Wnt canonical pathway in malignant plasma cells due to multiple mechanisms [8] and that this activation plays an important role in proliferation, survival, and drug resistance, so identifying the Wnt canonical pathway as a potentially attractive therapeutic target [9]. Tegavivint (BC2059), has been shown to exert potent cytotoxic effects against solid [10] and haematologic malignancies [11,12] including MM [9], with a predicted to be favourable toxicity profile and is currently in early phase clinical development (NCT03459469, NCT04851119, NCT04874480, NCT04780568). It exerts its activity by disrupting the binding of β-catenin to Transducin β-like protein 1 (TBL1) and its related protein (TBLR1), which under Wnt activation recognize and preferentially bind to hypoacetylated histones, thus localizing β-catenin to the Wnt target genes promoter [13,14].

In the present study we show that low concentrations of Tegavivint are able to potentiate the anti-MM effect of panobinostat in vitro, ex vivo, and in vivo including in both panobinostat and bortezomib resistant MM cells, thus providing the rational for the further evaluation of this potential novel therapeutic combination in RRMM.

## 2. Materials and Methods

### 2.1. Primary Samples Ex Vivo Treatment

Primary multiple myeloma samples were obtained from RRMM patients, following written informed consent with approval from the Alfred Hospital Research and Ethics Committee. Bone marrow mononuclear cells (BMMCs) were isolated with Ficoll-Paque Plus (Amersham Biosciences, Uppsala, Sweeden), washed and processed as previously described [9]. For further details see Supplementary Materials and Methods.

### 2.2. Myeloma Cell Lines

MM1R, NCIH929 and U266 Human Myeloma Cell Lines (HMCL) and HS5 (human fibroblasts) were obtained from the ATCC. OCIMy1, and IL6 dependent ANBL6 and XG1 were kind gifts from Frits Van Rhee (Winthrop P. Rockefeller Cancer Institute, Little Rock, AR, USA), whereas KMS34 was a kind gift from Kawasaki Medical School (Kurashiki, Japan) in 2008. HMCLs and HS5 cells were grown and treated at density 2.0 × 10^5^ cells/mL in RPMI1640 media (Gibco, Invitrogen, Waltham, MA, USA) supplemented with 10% heat-inactivated FBS (Lonza, Basel, Switzerland) and 2 mmol/L L-glutamine (Gibco, Invitrogen, Waltham, MA USA). 5 ng/mL of human IL6 (ProSpec-Tany TechnoGene, Ness-Ziona, Israel) was added in the medium for IL6 dependent cell lines ANBL6 and XG1.

### 2.3. Confocal Microscopy

OCIMy1 and U266 cells were treated with Tegavivint 100 nmol/L, panobinostat 10 nmol/L, Tegavivint and panobinostat or vehicle (DMSO) for 20 h and stained with TMRE (a fluorescent dye that is readily sequestered by active mitochondria thus acting as a marker of mitochondrial fitness) at 100 nmol/L (Sigma-Aldrich, Merck Pty. Ltd., Bayswater, Australia), Mitotracker Green FM (a green fluorescent mitochondrial stain which localizes to mitochondria regardless of mitochondrial membrane potential) at 200 nmol/L (ThermoFisher Scientific, Waltham, MA, USA) and Hoechst stain (ThermoFisher Scientific, Waltham, MA, USA) according to the manufacturer’s instructions. Carbonyl cyanide 4-(trifluoromethoxy)phenylhydrazone (FCCP) (a potent uncoupler of mitochondrial oxidative phosphorylation which disrupts ATP synthesis by transporting protons across the mitochondrial inner membrane) 1 μmol/L was used as a positive control. Fluorescence images were obtained by using an inverted Nikon A1r confocal microscope (Tokyo, Japan) equipped with a motorized piezo stage Galvano scanner and perfect focus system, using Apo LWD 40x WI λS DIC N2 (numerical aperture 1.15; Nikon, Tokyo, Japan). For further details see Supplementary Materials and Methods.

### 2.4. XF Mito Stress Analysis

OCIMy1, 2 × 10^5^ cells/mL, and U266 were plated and treated with DMSO, Tegavivint, panobinostat or their combination. At 18 h, cells were counted, centrifuged, and resuspended in the base medium containing 2 mM L-glutamine, 1 mM sodium pyruvate, and 10 mM glucose (Sigma-Aldrich, Merck Pty. Ltd., Bayswater, Australia) and seeded in a 96 well XF Cell Culture Microplate previously treated with Cell-Tak (Corning, Glendale, AZ, USA) according to the manufacturer’s instructions, at 5 × 10^4^ cells per well. Drugs were added accordingly. The oxygen consumption rate was measured using a XFe96 extracellular flux analyser (Agilent Technologies, Santa Clara, CA, USA) with sequential injection of 1 µmol/L oligomycin A (O), 1 µmol/L FCCP, and 1 µmol/L rotenone/antimycin A (R/A) (Sigma-Aldrich, Merck Pty. Ltd., Bayswater, Australia).

### 2.5. In Vivo Studies

Approval for the murine studies was obtained from the Animal Ethics Committee of the Alfred Hospital, Melbourne, Australia (E/1376/2031/M). Adult age-matched Cg-Prkdcscid Il2rgtm1Wjl/SzJ mice (The Jackson Laboratory, Ellsworth, ME, USA) were injected (intravenously) with 1 × 10^6^ U266 HMCL, carrying the FUL2-TG vector (a generous gift from Marco Herold, WEHI, Melbourne, Australia; with luciferase2 and GFP under the constitutively active ubiquitin and IRES promoter, respectively). Treatment was initiated at day 10 upon confirmation of established measurable disease by bioluminescence at day 7. Control mice (*n* = 5) received vehicle (17% Solutol HS 15 [Sigma-Aldrich, Merck Pty. Ltd., Bayswater, Australia] and normal saline), whereas treated mice received 30 mg/kg Tegavivint (*n* = 5) or 10 mg/kg panobinostat (*n* = 5) or their combination (*n* = 5) twice weekly intraperitoneally for 3 consecutive weeks for each of two 28 days cycles. Tumour burden was monitored on a weekly basis by in vivo imaging until the first mice reached experimental endpoint. Peripheral blood counts were sequentially monitored throughout the course of the experiment (Hemavet, Drew Scientific, Inc., Miami Lakes, FL, USA). Upon reaching the experimental endpoints (hind limb paralysis, >20% weight loss) the mice were humanely euthanized and tissues (spine and colon) were collected. Tissues were formalin fixed and embedded in paraffin, sectioned, and stained with H&E. Images were taken with an Olympus BX51 microscope.2.6. Micro-CT Studies

Images of the 5th lumbar vertebrae from the mice were acquired at the time of euthanasia using the Siemens Inveon PET/SPECT/CT scanner. The scans were performed using the computer tomography (CT) modality only, and the settings were as follows: voltage of 80 kV, current of 500 uA, 360 projections in step and shoot mode, 9.9 µm resolution reconstruction with a murine beam hardening algorithm applied. All reconstructions of the images were created with a reference HU factor for each sample ensuring the data was consistent. For µCT analysis see Supplementary Materials and Methods.

## 3. Results

### 3.1. Low Dose Combinations of Tegavivint and Panobinostat Exert Synergistic Anti-MM Effects In Vitro and Ex Vivo

**Figure 1:**
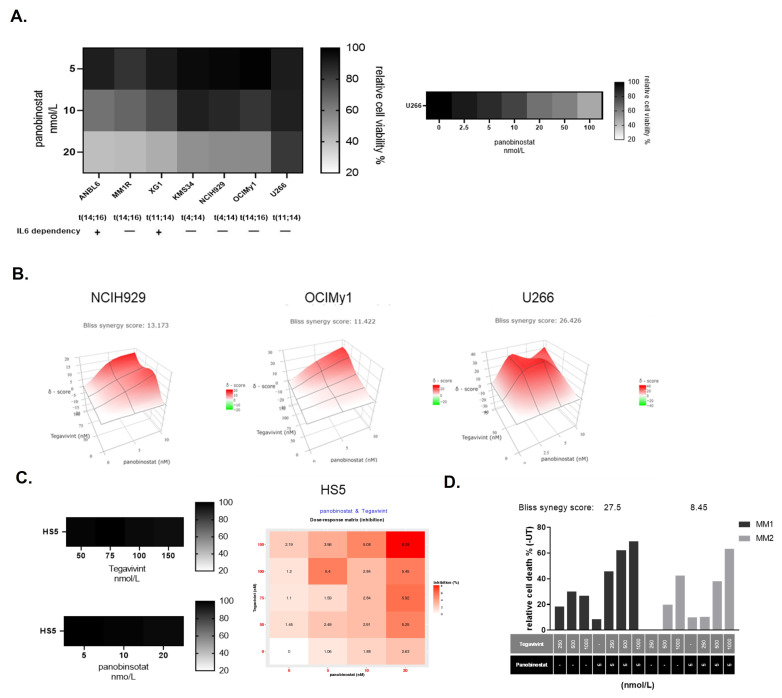
The combination of Tegavivint and panobinostat exerts synergistic anti-MM effects in vitro and ex vivo. (**A**) Seven HMCL were treated with clinically relevant doses of panobinostat (up to 20 nmol/L) for 48 h and PI+ was measured by FC (*n* = 3–6). Cell viability was calculated as a relative percentage compared with vehicle alone (100%). U266 were further treated with increasing doses of panobinostat up to 200 nmol/L for 48 h and propidium iodide + (PI+) was measured by flow cytometry (FC) (*n* = 6). (**B**) NCIH929, OCIMy1, and U266 were treated with Tegavivint, panobinsotat, or their combination for 48 h. PI+ was measured by FC (*n* = 3). The expected drug combination responses were calculated based on Bliss reference model using SynergyFinder. Deviations between observed and expected responses with positive and negative values denote synergy and antagonism respectively. (**C**) Non-malignant HS5 cells were treated with similar doses of Tegavivint or panobinostat alone for 48 h and PI+ was measured by FC. Cell viability was calculated as a relative percentage compared with vehicle alone (100%) (*n* = 3) (left panel). HS5 cells were treated with Tegavivint, panobinsotat, or their combination for 48 h and PI+ was measured by flow cytometry (FC) (*n* = 3). Dose response matrix was produced (right panel). (**D**) Proportion of apoptotic (Apo 2.7 +) CD38^+^ CD45^−^ primary MM cells in an autologous bone marrow coculture assay after 72 h of Tegavivint, panobinostat, or the combination. Numbers represent calculated Bliss synergy scores for each patient sample.

HMCL have variable sensitivity to HDACi, including panobinostat, with the t(11;14) positive HMCL U266 being reported as highly resistant to the latter [15]. We determined the effect of panobinostat on the viability of seven genetically heterogeneous HMCL, including U266 and IL6 dependent ANBL6 and XG1, at 48 h (Figure 1A, left panel). Addition of IL6 did not influence the resistance of HMCL to panobinostat. U266 proved to be the most resistant cell line, with an LD_50_ = 81.3 nmol/L (Figure 1A, right panel), whereas OCIMy1 [t(14;16) positive] and NCIH929 [t(4;14) positive] showed intermediate sensitivities with LD_50_ of 31.9 and 41.8 nmol/L, respectively (Supplementary Figure S1A). All three HMCL have been previously shown to have intermediate sensitivity to Tegavivint, with LD_50_ at 24 h around 200 nmol/L [9]. Their dose response to Tegavivint at 48 h was further validated (Supplementary Figure S1B) and combination treatment with low concentrations of panobinostat (≤10 nmol/L) and Tegavivint (≤150 nmol/L) were evaluated at 48 h. In all three HMCL the combination proved to be highly synergistic, particularly U266 which exhibited the highest synergy score [16] (Bliss synergy score U266 = 26.4, NCIH929 = 13.2, OCIMy1 = 11.4) (Figure 1B). In contrast panobinostat or Tegavivint alone at the same doses did not affect the viability of non-malignant HS5 cells (Figure 1C, left panel). In accordance, combination of the two drugs in HS5 did not induce significant cell death (max relative cell death: 8.28% with panobinostat 20 nmol/L and Tegavivint 150 nmol/L) (Figure 1C, right panel).

The effect of the combination on ex vivo primary MM cells was subsequently evaluated. Primary MM tumour cells from two patients with RRMM were treated for 72 h, after which the CD38+ CD45− MM cell population was assessed for apoptosis by flow cytometry (Figure 1D). In accordance with the in vitro results the combination of Tegavivint with panobinostat proved to be synergistic with calculated Bliss synergy scores 27.5 and 8.42 respectively (Supplementary Figure S2A). In contrast, the combination had no synergistic pro-apoptotic effect to the non-myeloma stromal cells, with calculated Bliss synergy scores −0.59 and −2.66 respectively (Supplementary Figure S2B).

### 3.2. The Combination of Tegavivint and Panobinostat Induces Apoptosis and Downregulates Wnt Pathway Down-Stream Targets

**Figure 2:**
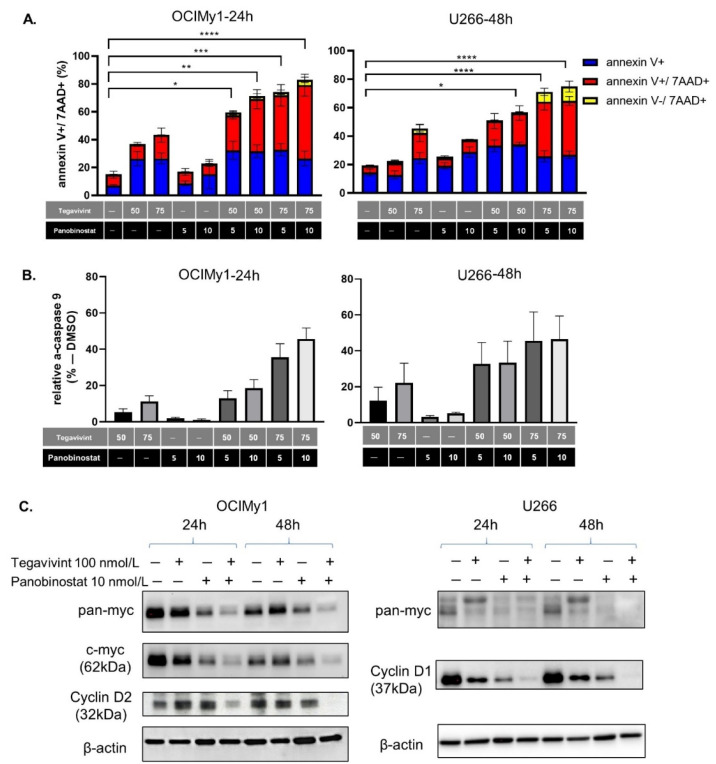
The combination of Tegavivint and panobinostat induces apoptotic cell death and down-regulation of β-catenin targets. (**A**) Percentage of annexin V, annexin V/7AAD, and 7AAD-positive cells after Tegavivint, panobinostat or the combination for OCIMy1 and U266 at 24 and 48 h respectively (*n* = 3, ±SE). 2way ANOVA Dunett’s multiple comparisons test of row means to control row mean (DMSO treated) (* *p* = 0.0175, ** p = 0.0013, *** *p* = 0.0007, **** p < 0.0001 for OCIMy1 and * *p* = 0.0164, **** *p* < 0.0001 for U266). (**B**) Percentage of active caspase-9 after treatment with Tegavivint, panobinostat, or the combination for OCIMy1 and U266 at 24 and 48 h respectively relative to the vehicle (DMSO) treated cells (*n* = 3, ±SE). (**C**) Immunoblotting of whole-cell lysates for β-catenin down-stream targets Myc, Cyclin D1 and Cyclin D2 after 24 or 48 h of treatment with Tegavivint, panobinostat, or the combination for OCiMy1 and U266. Loading control: β-actin (*n* = 3). Detailed information about the Western blotting can be found at Figure S4.

We quantified apoptosis induction in OCIMy1 and U266 by annexin V, annexin V/7AAD to the combination of Tegavivint (50–75 nmol/L) and panobinostat (5–10 nmol/L). Apoptosis initiation and commitment of cells to late apoptosis (annexin V+/7AAD+) were apparent for OCIMy1 at 24 h whereas for U266 they were significantly augmented at 48 h (Figure 2A, Supplementary Figure S3A), while there was no significant increase of annexin V−/7AAD+ cells (indicative of non-apoptotic cell death). These findings were consistent with apoptotic activation being one of the main mediators of cell death induced by the combination. Accordingly, the levels of active caspase 9 (a measure of the intrinsic apoptotic pathway activity) were elevated at the same timepoints (46% for OCIMy1 treated with 75 nmol/L of Tegavivint and 10 nmol/L panobinostat, and 47% for U266 treated with the same combination) (Figure 2B, Supplementary Figure S3B).

Cyclin D and Myc dysregulation plays a critical role in the pathogenesis of MM [17] and both are down-stream targets of the canonical Wnt pathway [11]. Combination treatment of OCIMy1 (expressing high Myc and Cyclin D2 at baseline) and U266 (expressing high L-Myc and Cyclin D1 at baseline) induced significant Myc and Cyclin D down-regulation as early as 24 h after treatment (Figure 2C, Supplementary Figure S4).

Collectively, our results confirm the synergistic pro-apoptotic effect of the combination and its significant negative impact on the expression of MM promoting oncogenes.

### 3.3. Tegavivint in Combination with Panobinostat Targets the Mitochondria Fitness of MM Cells

There is an increasing body of evidence implicating Myc in the metabolic reprogramming of cancer cells [18,19] whereas HDACi have been shown to alter glucose metabolism in MM cells [20]. In this context, having shown down-regulation of Myc protein expression with the combination of Tegavivint and panobinostat, we evaluated their effect on the mitochondrial fitness and metabolic activity of OCIMy1 and U266 cells.

**Figure 3:**
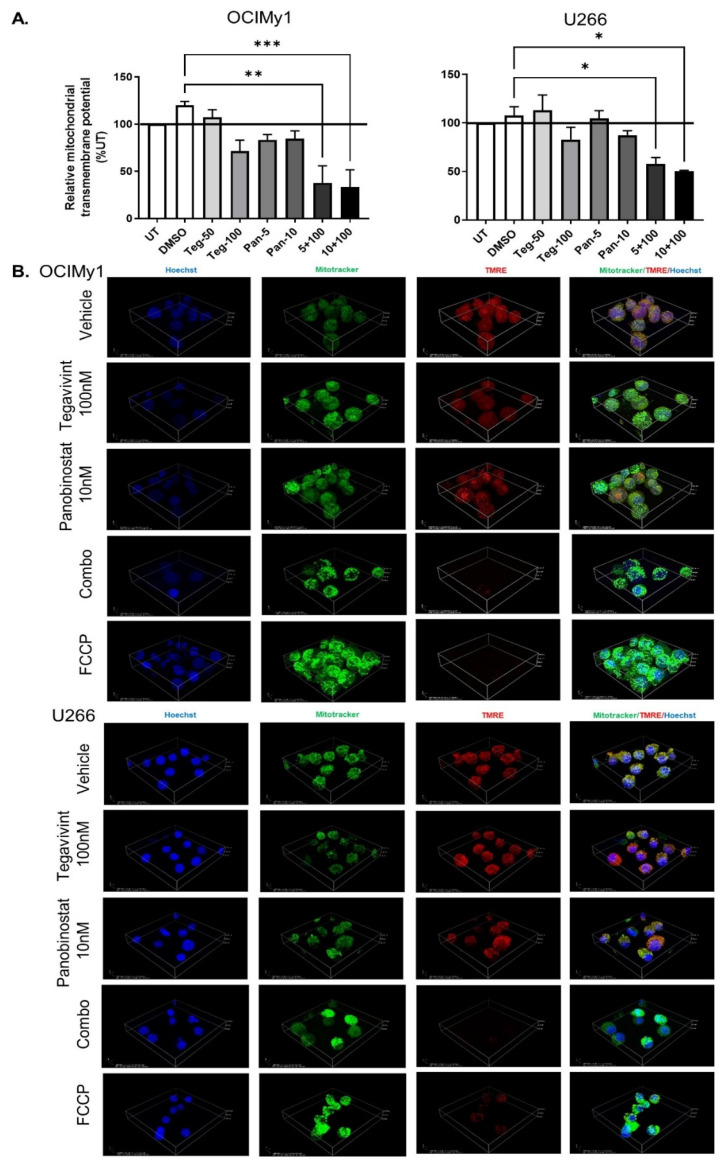
Tegavivint in combination with panobinostat targets mitochondrial fitness of MM cells. (**A**) OCIMy1 and U266 cells were treated with Tegavivint (Teg-), panobinostat (Pan-), Tegavivint and panobinostat, or vehicle (DMSO) for 20 h. Mitochondrial transmembrane potential was measured by TMRE staining and FC. MFI was measured and expressed as a relative % to the TMRE MFI of UT (untreated) cells (UT = 100%) (*n* = 3, ±SE, nonparametric Kruskal-Wallis multiple comparisons test, ** *p* = 0.0013, *** *p* = 0.001 for OCIMy1, and * *p* = 0.04 and 0.03 for U266 respectively). (**B**) OCIMy1 and U266 were treated with Tegavivint, panobinostat or the combination for 20 h and stained with TMRE (red for mitochondrial potential), Mito Tracker Green (green for mitochondria) and Hoechst 33,342 (blue for nuclei) and evaluated with confocal microscopy. One μmol/L of the uncoupling agent FCCP was added 20 min before imaging and used as a positive control (magnification 40× WI).

The MFI (median fluorescent intensity) of TMRE (measure of transmembrane potential/mitochondrial fitness) fell significantly upon the addition of 100 nmol/L Tegavivint with 5 or 10 nmol/L of panobinostat for 20 h (*p* = 0.001 and *p* = 0.001, and *p* = 0.05 and *p* = 0.03, for OCIMy1 and U266, respectively) (Figure 3A). Consistent with this observation, analysis of TMRE by confocal microscopy in combination with MitoTracker Green FM staining similarly demonstrated the deleterious impact of the combination on the mitochondria fitness of both cell lines (Figure 3B, Supplementary Figure S5A). Although Wnt signalling and consequently β-catenin have been shown to strongly activate mitochondrial biogenesis [21,22] the combination of Tegavivint and panobinostat did not numerically alter the mitochondria, as measured by MitoTracker Green FM staining and FC, during the time frame of the described experiments (Supplementary Figure S5B).

**Figure 4:**
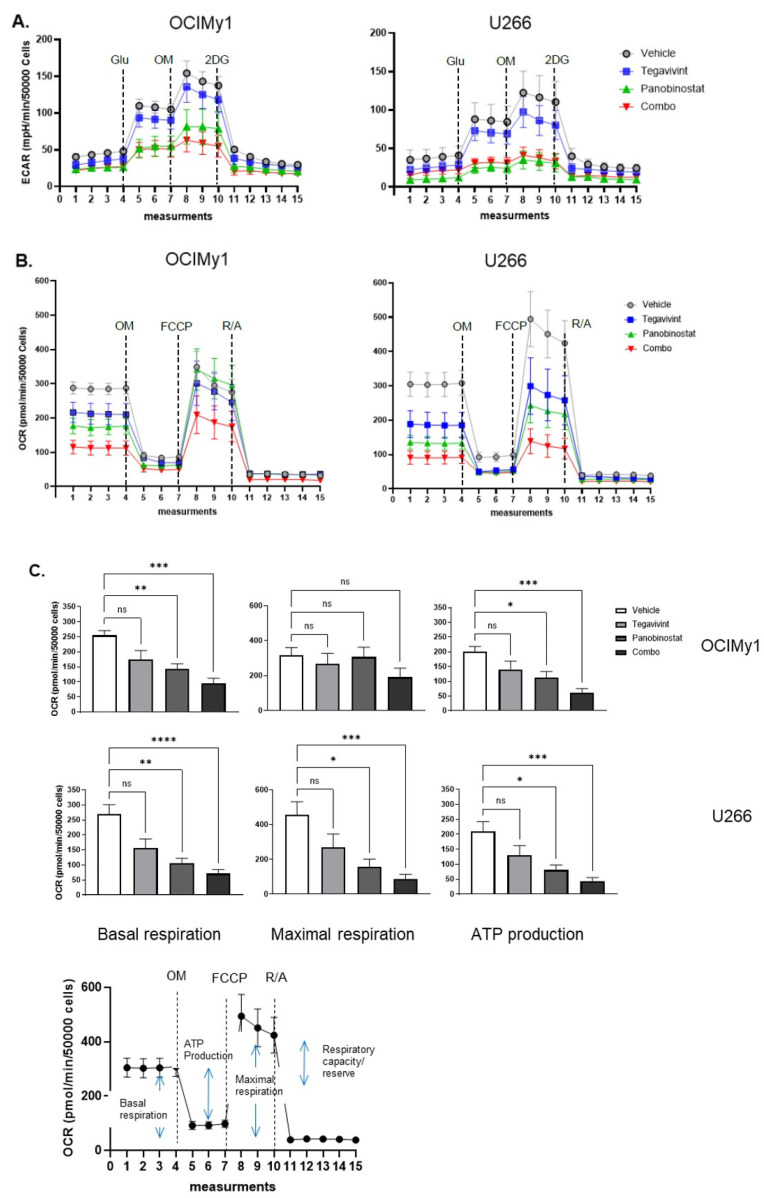
Tegavivint in combination with panobinostat reduces oxidative phosphorylation of MM cells. (**A**) OCIMy1 and U266 cells were treated with vehicle, 100 nmol/L of Tegavivint, 10 nmol/L of panobinostat, or the combination for 18 h. Analysis of ECAR (extracellular acidification rate) was performed using a Seahorse XF analyser to assess glycolysis in the context of a glycolysis stress test. Three technical replicates were performed for each experimental variable (*n* = 3, ±SE) (Glu: glucose, OM: oligomycin, 2DG: 2-deoxy-D-glucose). (**B**) OCIMy1 and U266 cells were treated similarly to Figure 3A for 18 h. Analysis of OCR (oxygen consumption rate) was performed using a Seahorse XF analyser to assess mitochondrial respiration/oxphos (oxidative phosphorylation) in the context of a mitochondrial stress test. Three technical replicates were performed for each experimental variable (*n* = 3, ±SE) (OM: oligomycin, FCCP: carbonyl cyanide 4-(trifluoromethoxy)phenylhydrazone R/A: rotenone/antimycin). (**C**) Results from the OCR analysis were further analysed and the basal respiration, maximal respiration, and ATP production for OCIMy1 and U266 were calculated (*n* = 3, ±SE) (nonparametric Kruskal–Wallis multiple comparisons test * *p* ≤ 0.05, ** *p* ≤ 0.01, *** *p* ≤ 0.001, **** *p* ≤ 0.001).

To further study the effect of Tegavivint and panobinostat on cellular metabolism we conducted extracellular flux analysis. We assessed the effect of single drugs (Tegavivint 100 nmol/L and panobinostat 10 nmol/L) and the combination on glycolysis by determining ECAR (extracellular acidification rate) in the context of a glycolysis stress test (Figure 4A). Consistent with the known effects of HDACi on aerobic glycolysis [20] panobinostat was able to significantly suppress glycolysis in both cell lines (measures derived from the measurement of ECAR) as a single agent, with the effect further augmented by the addition of Tegavivint in terms of glycolytic capacity (Supplementary Figure S6). Knowledge on the effects of Myc and/or panobinostat treatment on oxidative phosphorylation (oxphos) is limited. To this end, we assessed the effect of single drugs and the combination on oxphos by measuring the OCR (oxygen consumption rate) in the context of a mitochondrial stress test (Figure 4B). This revealed that both basal respiration and ATP production were significantly reduced by the combination treatment—*p* = 0.0001 and *p* = 0.0001, respectively, for OCIMy1, and *p* < 0.0001 and *p* = 0.0004, respectively, for U266. Moreover, for U266, which demonstrated higher respiratory capacity, maximal respiration was also significantly reduced by the combination (*p* = 0.0002) (Figure 4C). Collectively, these data demonstrate that in MM cells the combination of Tegavivint and panobinostat markedly inhibits oxidative phosphorylation and to a lesser extent aerobic glycolysis thus impairing metabolic pathways essential for energy and biomolecule generation.

### 3.4. Tegavivint Overcomes Acquired Panobinostat Resistance in Secondary Bortezomib Resistant MM Cells

**Figure 5:**
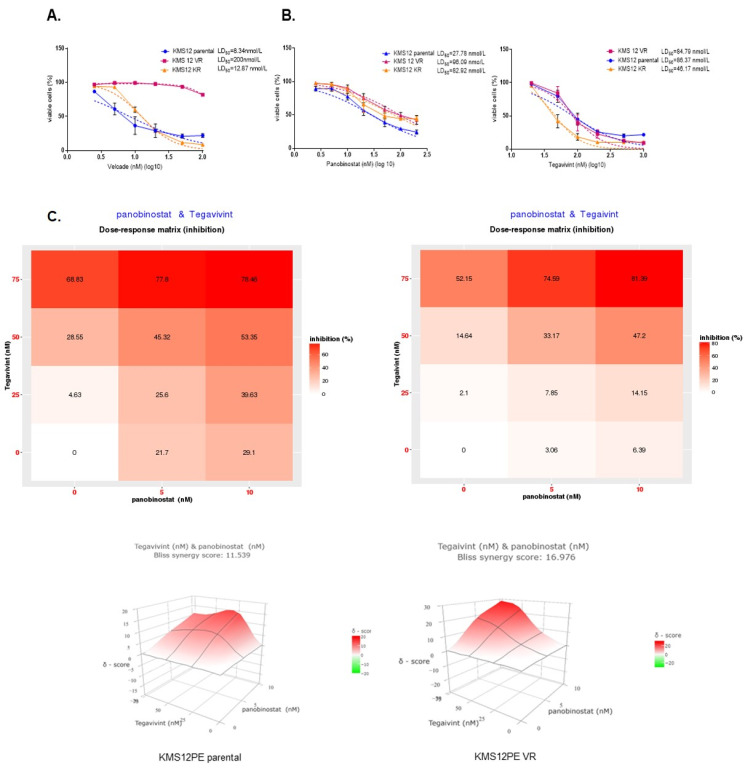
Tegavivint overcomes acquired panobinostat resistance in bortezomib resistant MM cells. The HMCL KMS12PE was cultured for a 12 months period in slowly increasing concentrations of bortezomib (VR), carfilzomib (KR), or vehicle (parental). Resistant cells were stored for 6 months, thawed and cultured for an additional 12 week period in the absence of drugs to confirm the acquisition of permanent drug resistance. (**A**). Cells were treated with increasing doses of bortezomib or carfilzomib for 48 h and cell viability was calculated after PI staining and FC (% of vehicle treated cells). (**B**). Parental, VR and KR cells were treated with increasing doses of panobinostat for 48 h and cell viability was calculated after PI staining and FC (% of UT) (left panel). Parental, VR and KR cells were treated with increasing doses of Tegavivint for 48 h and cell viability was calculated after PI staining and FC (% of UT) (right panel). For calculations of LD_50_ we applied the non-linear regression model. Dashed lines correspond to the non-linear fit curves. (**C**). Parental and VR cells were treated with Tegavivint, panobinostat or the combination for 48 h and the PI+ was measured by FC (*n* = 3). Dose response matrices were produced for both cell lines and the expected drug combination responses were calculated based on the Bliss reference model using SynergyFinder. Deviations between observed and expected responses with positive and negative values denote synergy and antagonism respectively.

A recent meta-analysis evaluating the efficacy and safety of HDACi, including panobinostat, in RRMM, demonstrated that patients who were bortezomib-refractory were less likely to benefit from HDACi [23]. To investigate the effect of Tegavivint and panobinostat in the context of proteasome-inhibitor (PI) resistance we generated KMS12PE cells resistant to both carfilzomib (KR) and bortezomib (VR). Although the KR and parental cells both remained sensitive to bortezomib (LD_50_ = 8.3 nmol/L and 12.9 nmol/L, respectively), the VR cell line showed a 20-fold increase in bortezomib LD_50_ (LD_50_ = 200 nmol/L) confirming the acquisition of secondary bortezomib resistance (Figure 5A). PI resistance was accompanied by relative panobinostat resistance in both resistant cell lines (LD_50_ = 96.1 nmol/L for VR and 82.9 nmol/L for KR vs. LD_50_ = 27.8 nmol/L for the parental cells) (Figure 4B—left panel). Although there was no difference of sensitivity to Tegavivint amongst parental and VR cells (LD_50_ = 84.8 nmol/L vs. LD_50_ = 86.4 for the VR and parental cells, respectively), KR cells showed a significant increase of sensitivity (LD_50_ = 46.2 nmol/L) (Figure 5B—right panel). Importantly, Tegavivint at concentrations ranging from 25 nmol/L to 75 nmol/L was able to re-sensitise VR cells to low panobinostat concentrations (5 and 10 nmol/L), and further augment the synergistic effect of the two drugs as shown by an increased Bliss synergy score (17.0) compared to the corresponding score for the parental cells (12.0) (Figure 5C).

These results suggest that the combination of Tegavivint and panobinostat may be able to overcome the therapy resistant phenotype in RRMM patients with primary or secondary bortezomib refractoriness.

### 3.5. Tegavivint in Combination with Panobinostat Exerts Potent Anti-MM Effects In Vivo

**Figure 6:**
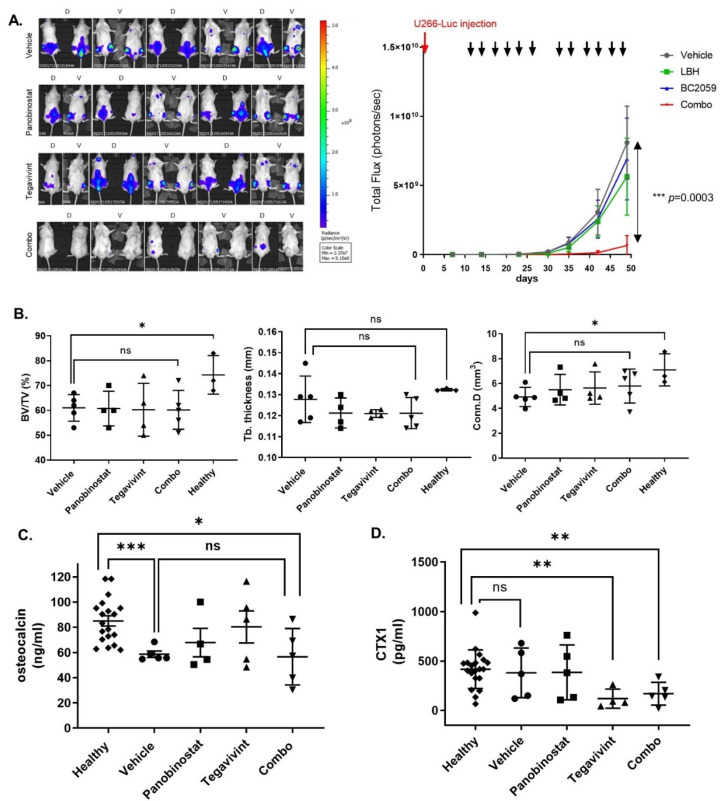
Tegavivint in combination with panobinostat exerts potent anti-MM effect in vivo, without a negative impact on bone metabolism. (**A**) NSG human MM-bearing mice received vehicle (17% Solutol HS 15 (Sigma-Aldrich) and normal saline) (*n* = 5), whereas treated mice received 10 mg/kg panobinostat (*n* = 5) or 30 mg/kg Tegavivint (*n* = 5), or their combination (*n* = 5) twice a week intraperitoneally for 3 consecutive weeks for 2 cycles of 28 days each. Tumour growth was monitored weekly with in vivo bioluminescence imaging. Dorsal (D) and ventral (V) views of NSG human MM-bearing mice at day 49 (end of cycle 2 treatment). The median flux (photons/second) of dorsal and ventral views for each cohort is documented (*n* = 5 for each cohort, ±SD). Multiple *t*-tests were used for statistical analysis without assuming SD, and statistical significance was determined using the Holm-Sidak method, with α = 5.000%. ↓: therapeutic intervention. (**B**) Adult NSG human MM-bearing mice were treated as mentioned above for two cycles. Upon reaching experimental end point mice were humanely euthanised and micro-CT of L5 vertebrae was performed. BoneJ was used for the analysis and the calculation of bone morphometric indices and compared with the measurements derived from three healthy age matched NSG mice. Treatment with single drugs or the combination did not negatively affect the bone volume fraction (BV/TV), trabecular thickness (Tb. Thickness) or the connectivity density (Conn. D) (* *p* ≤ 0.05, nonparametric Mann-Whitney *t* test). (**C**) During the last week of cycle 2 serum was analysed for osteocalcin levels by ELISA. Derived values were compared with serum osteocalcin concentrations before the injection of U266-MM cells (healthy) (*** *p* = 0.0005 and * *p* = 0.02, nonparametric Mann-Whitney *t* test). (**D**) Similarly, serum was analysed for carboxy-terminal collagen crosslinks (CTX1) levels by ELISA. Derived values were compared with serum CTX1 concentrations before the injection of U266-MM cells (healthy) (** *p* = 0.005 and ** *p* = 0.008 for healthy versus Tegavivint or combination, respectively, nonparametric Mann-Whitney *t*-tests).

We assessed the in vivo activity of low dose Tegavivint and panobinostat both as single agents and in combination using a biweekly dosing schedule, for both panobinostat and Tegavivint, in a systemic myelomatosis xenograft utilising U266 cells, for a total of two cycles. Panobinostat was administered at 10 mg/kg biweekly, while the approved dose for the treatment of patients with RRMM is 20 mg/kg, three times weekly [5]. Tegavivint was administered at 30 mg/kg, while 50 mg/kg have been shown to exert anti-tumour effect in a solid tumour mouse model [10]. The animals were treated for the first three weeks of each of two 28-day cycles, so, three weeks on one week off. With the combination, significant inhibition of tumour growth, as measured by luciferase bioluminescence, was evident by day 14 of cycle 1 (*p* = 0.02). After the last administration of drugs (day 49) (Figure 6A left panel) the reduction in tumour burden with the combination when compared to vehicle control, Tegavivint, or panobinostat was highly statistically significant (*p* = 0.0003) (Figure 6A right panel). Moreover, and despite the limited number of therapeutic cycles the inhibition of tumour growth induced by the combination translated into a significant prolongation in survival (*p* = 0.01) (Supplementary Figure S7).

### 3.6. Tegavivint in Combination with Panobinostat Is Well Tolerated In Vivo

The pivotal role of the Wnt canonical pathway in haemopoietic stem cell (HSC) maintenance [24] has raised theoretical concerns in relation to the therapeutic applicability of Wnt pathway inhibitors. In our in vivo experiment biweekly dosing for two cycles while exhibiting significant anti-MM activity was not associated with weight loss (Supplementary Figure S8A, left panel) consistent with the maintenance of intact gastro-intestinal function. Furthermore, immune-histochemical (IHC) studies of the colon harvested at the time of euthanasia demonstrated normal histologic features in the combination treated mice (Supplementary Figure S8A, right panel). Moreover, combination was not associated with peripheral blood cytopenias at any of the evaluation time-points (Supplementary Figure S8B).

### 3.7. Tegavivint in Combination with Panobinostat Does Not Negatively Impact Bone Metabolism In Vivo

The Wnt pathway is recognised as being a key regulator of bone homeostasis [25]. This is of particular relevance in MM as almost 80% of newly diagnosed MM patients have evidence of significant bone loss due to dysregulation of complex and not yet fully understood interactions between osteoblasts, osteoclasts, osteocytes, bone matrix, and immune cells [26]. Considering the engraftment of U266 in the bone marrow of the spine (especially lumbar) and long bones (Figure 6A) and in order to study the potential impact of Tegavivint on bone homeostasis, we performed micro-CT of the 5th lumbar vertebrae from the xenografts harvested at the time of euthanasia. Micro-CT results were compared to those from three healthy control NSG mice of similar age. While the presence of MM disease was associated with a significant decrease in bone volume fraction BV/TV (BV: bone volume, TV: total volume of interest) (*p* = 0.04) no additive bone loss was observed in the animals treated with Tegavivint or with Tegavivint in combination with panobinostat (Figure 6B).

Serum levels of osteocalcin (a measure of osteoblastic activity) and CTX1 (carboxy-terminal collagen crosslinks) (a measure of osteoclastic activity) were measured at baseline (before the injection of MM cells) (healthy) and at the last week of treatment of cycle 2. A reduction in osteocalcin (healthy vs. vehicle, *p* < 0.001), representative of MM-induced inhibition of osteoblastic activity was consistent with the micro-CT findings but importantly there was no evidence of additional inhibition of osteoblastic activity related to Tegavivint (Figure 6C). Interestingly, CTX1 levels at the same time point showed a significant decrease in osteoclastic activity in the Tegavivint alone and Tegavivint and panobinostat combination treated animals when compared to the healthy control levels, the mechanism of which is unclear (Figure 6D).

## 4. Discussion

Panobinostat is the only HDACi approved in the USA and Europe for the treatment of RRMM [5]. Approval was based on the results of the PANORAMA 2 TRIAL which paradoxically demonstrated a relatively greater survival benefit for the most heavily pretreated patients receiving panobinostat in combination with intra-venous bortezomib and dexamethasone, likely reflecting the unique and pleiotropic effects of HDACi on MM cells [4]. The combination was, however, associated with a challenging toxicity profile that is largely mitigated with the use of sub-cutaneous bortezomib and alternative panobinostat scheduling as demonstrated in the recently published PANORAM 3 trial [5]. This fact, notwithstanding the role and optimal combinatorial approach for panobinostat use in MM, remains unclear.

Several lines of evidence have confirmed the presence of an active intrinsic Wnt canonical signalling in MM [8] as a result of both genetic and epigenetic abnormalities [23], with greater dysregulation evident, coincident with disease progression [27]. Significantly, upregulation of the Wnt pathway is also thought to be an important mechanism driving lenalidomide resistance [28] further supporting the exploration and validation of Wnt pathway inhibitors for the treatment of RRMM. Interactions of β-catenin-HDAC have long been recognised [29,30], and a dual targeting approach has been successfully validated in AML [11]. In this work we showed that low doses of the β-catenin inhibitor Tegavivint can augment the anti-MM effect of low dose panobinostat, by initiating intrinsic apoptosis, even in the highly resistant U266. This cell line despite carrying t(11;14), which recently has been shown to respond to Bcl2 inhibitor venetoclax [31], is resistant to the latter [32], rendering it a difficult to kill cell line. Dual inhibition was able to significantly down-regulate known targets of the Wnt canonical pathway, namely Cyclin D1, Cyclin D2, and Myc. Myc is a well-recognised pro-survival factor and master regulator of glycolysis [18], whereas HDAC inhibitors have been shown to affect aerobic glycolysis [20]. Accordingly, panobinostat, was able to reduce the aerobic glycolytic activity of MM cells, as measured by ECAR, without though inducing significant cell death, whereas only the combination was able to negatively impact oxidative phosphorylation, measured by OCR. The significant decrease of OCR and loss of mitochondrial transmembrane potential was shown to be due to mitochondrial damage and not due to decreased mitochondria biogenesis. Thus, combination of both drugs was capable of compromising both energy production machineries leading to MM cell apoptotic cell death.

Bortezomib refractory patients were only included in the PANORAMA 2 study while excluded in the PANORAMA 1 and 3 [5]. A meta-analysis of panobinostat treated patients showed an overall response rate of 32% for those refractory to bortezomib and 43% for those refractory to lenalidomide suggesting that panobinostat had superior activity in the latter subgroup [22]. In our study we were able to confirm the acquisition of panobinostat resistance upon acquiring bortezomib or carfilzomib resistance in vitro and that the addition of Tegavivint was able to reverse panobinostat resistance in the former, underpinning the adoption of alternative combination for the treatment of patients with PI resistance. The mechanisms of panobinostat resistance in these cell lines are currently being investigated.

One of the aims of the recently published PANORAMA 3 was to assess alternative scheduling and dosing of panobinostat to mitigate its toxicity. Although the regimen with the highest dose and frequency was the most effective, lower doses (10 mg) proved to be better tolerated. Similarly, for the in vivo validation of the proposed combination we used low dose panobinostat (10 mg) administered twice weekly in combination with low dose Tegavivint with no significant toxicity seen after the completion of two cycles of treatment. Our scheduling, as shown by the tumour growth inhibition benefit, was able to exert significant anti-tumour effect while minimising potential toxicity.

The central role of the Wnt canonical pathway in orchestrating homeostasis of various tissues, including adult bone, has hindered the development of Wnt pathway inhibitors. Interestingly, the results of several studies evaluating monoclonal antibodies and small molecule inhibitors in a variety of Wnt driven tumours have been promising, with the majority of these molecules, including Tegavivint, being well tolerated in the models utilised [9,10,11,12]. The lack of toxicity is believed to be due to the ‘just-right’ model of Wnt pathway activation, which suggests that each tissue, including tumour cells, have an optimal and different threshold of Wnt activation [23,33]. Accordingly, in our in vivo model the combination treatment did not negatively affect haemopoiesis, GI tract epithelium, or bone homeostasis validated at the end of the experiment (different time point for each mouse). Additionally, at these low dosages Tegavivint alone or in combination with panobinostat at the end of the 2nd cycle did not further inhibit osteoblastic activity but intriguingly down-regulated osteoclastic activity. The reduced osteoclastic activity cannot be explained by differences in tumour burden as tumour growth in the Tegavivint alone cohort did not differ significantly from the vehicle cohort. One possible explanation could be decreased secretion of DKK1 by MM cells. DKK1, a Wnt pathway inhibitor is a downstream target of β-catenin [34], known to promote osteoclastic activity, while inhibiting osteoblastic differentiation [35], thus chemical inhibition of the pathway could lead to down-regulation of its transcription, but this hypothesis would require further evaluation.

It is well known that the Wnt signaling pathway has been implicated in multiple aspects of MM disease, namely disease progression and acquisition of drug resistance as well as imbalance between bone-forming osteoblasts and bone-resorbing osteoclast, leading to the characteristic osteolytic bone lesions of MM patients [8]. Thus, targeting the Wnt pathway could prove to be an attractive avenue, especially for patients who have acquired resistance to mainstream therapies, without further deteriorating their bone disease, as concluded from our in vivo experiment. Nevertheless, taking into consideration the central role of the Wnt pathway in regulating tissue-specific stem cell populations [36], close monitoring of blood counts and GI tract condition is suggested for patients with RRMM receiving the aforementioned combination.

## 5. Conclusions

Novel drugs and drug combinations have undoubtfully changed the paradigm for the treatment of MM, leading to significant prolongation of both PFS and OS. Nonetheless, the majority of patients will sequence through multiple relapses exhausting all possible treatments, through the acquisition of drug resistance to the mainstream therapies. We have shown in this study that panobinostat, in combination with Tegavivint has a significant anti-MM effect with a favourable toxic profile and may prove to be a beneficial alternative for the treatment of highly pre-treated RRMM that warrants further evaluation.

## Data Availability

The data presented in this study are available on request to the corresponding authors.

## Supplementary Materials

The following are available online at https://www.mdpi.com/article/10.3390/cancers14030840/s1, Figure S1: (A) NCIH929 and OCIMy1 HMCL were treated with increasing doses of panobinostat for 48 h and cell viability was calculated after PI staining and FC, (B) NCIH929, OCIMy1 and U266 HMCL were treated with increasing doses of Tegavivint for 48 h and cell viability was calculated after PI staining and FC, Figure S2: (A) Calculation of Bliss synergy score for primary MM cells in an autologous bone marrow coculture assay after 72 h of Tegavivint, panobinostat, or their combination (B) Calculation of Bliss synergy score for primary stromal cells in the aforementioned experiment, Figure S3: (A) Percentage of annexin V, annexin V/7AAD, and 7AAD positive cells after vehicle alone (DMSO), Tegavivint (50 and 75 nmol/L), panobinostat (5 and 10 nmol/L), and combination treatment of U266 for 24 h (*n* = 3, ±SE), (B) Percentage of active caspase-9 after treatment with Tegavivint, panobinostat, or the combination for OCIMy1 and U266 at 24 and 48 h respectively (raw data), Figure S4: Composite images (chemiluminescent and colorimetric)/scanned images (cyclin D2) derived from immunoblotting of OCIMy1 and U266 whole-cell lysates for β-catenin down-stream targets Myc, Cyclin D1, and Cyclin D2 after 24 or 48 h of treatment with Tegavivint, panobinostat, or their combination, Figure S5: (A) Larger view of OCIMy1 treated with DMSO for 20 h, stained with TMRE (red for mitochondrial potential), Mito Tracker Green (green for mitochondria) and Hoechst 33342 (blue for nuclei) and evaluated with confocal microscopy, (B) OCIMy1 and U266 were treated with 100 nmol/L of Tegavivint in combination with 10 nmol/L of panobinostat or vehicle (DMSO) for 20 h. Mitochondrial load was measured by Mitotracker Green straining and FC, Figure S6: OCIMy1 and U266 were treated with vehicle, 100 nmol/L of Tegavivint, 10 nmol/L of panobinostat, or combination for 18 h. Analysis of ECAR was performed using Seahorse XF analyser to assess glycolysis in the context of a glycolysis stress test, Figure S7: NSG human MM-bearing mice were treated with vehicle alone, 10 mg/kg of panobinostat, 30 mg/kg of Tegavivint, or the combination of panobinostat and Tegavivint, Kaplan–Meier survival curves were calculated, Figure S8: (A) Panobinostat alone, Tegavivint alone or combination treatment did not affect the GI tract of of the MM-bearing mice as validated by body weight (BW) monitoring (left panel) and IHC of colonic mucosa (right panel). (B) At completion of two treatment cycles (day 57) blood was collected and cell counts were performed. References [37,38] are cited in the supplementary materials.
