# Supplementary material for: Combination of Histone Deacetylase Inhibitor Panobinostat (LBH589) with β-Catenin Inhibitor Tegavivint (BC2059) Exerts Significant Anti-Myeloma Activity Both In Vitro and In Vivo
Source: Cancers (Basel). 2022 Feb 8;14(3):840. doi: 10.3390/cancers14030840 (PMC8834319; doi:10.3390/cancers14030840)
Supplement: Supplementary file 1 [file cancers-14-00840-s001.zip › cancers-1572119-supplementary.pdf]

Supplementary Materials

Combination of Histone Deacetylase Inhibitor Panobinostat (LBH589) with β-Catenin Inhibitor Tegavivint (BC2059) Exerts Significant Anti-Myeloma Activity Both In Vitro and In Vivo

Ioanna Savvidou, Tiffany Khong, Sophie Whish, Irena Carmichael, Tara Sepehrizadeh, Sridurga Mithraprabhu, Stephen K. Horrigan, Michael DeVeer and Andrew Spencer

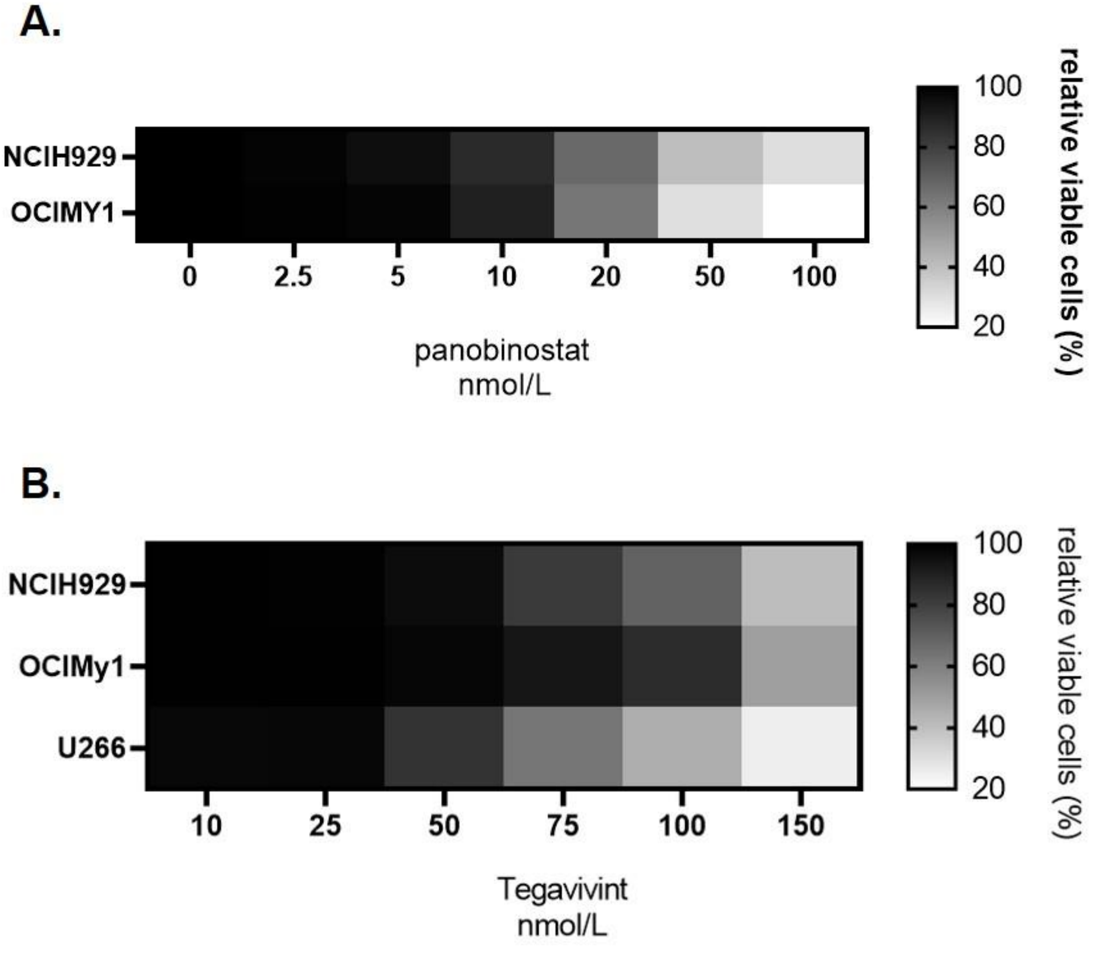

Figure S1. (**A**) NCIH929 and OCIMy1 HMCL were treated with increasing doses of panobinostat for 48 hours and cell viability was calculated after PI staining and FC (relative % to vehicle treated cells — 0 nmol/L) (*n* = 3–5). (**B**) NCIH929 OCIMy1 and U266 HMCL were treated with increasing doses of Tegavivint for 48 hours and cell viability was calculated after PI staining and FC (relative % to vehicle treated cells) (*n* = 3–6).

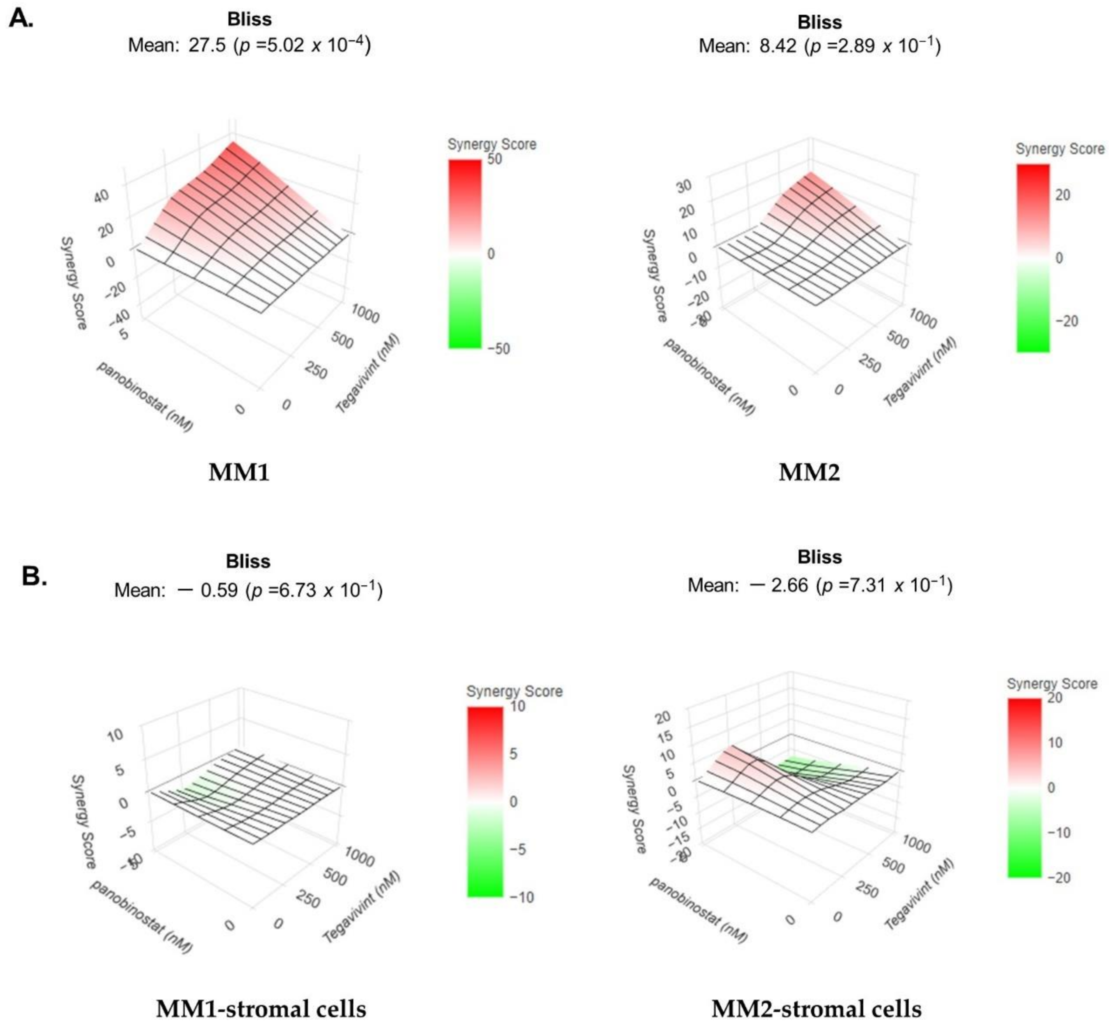

Figure S2. (**A**) Proportion of apoptotic (Apo 2.7 +) $CD38^{+}$/ $CD45^{-}$ primary MM cells in an autologous bone marrow coculture assay after 72 hours of Tegavivint, panobinostat or the combination was validated. The expected drug combination responses were further calculated based on Bliss reference model using SynergyFinder. Deviations between observed and expected responses with positive and negative values denote synergy and antagonism respectively. (Due to the use of a single dose of panobinostat, synergy score calculation and interpretation has limitations). (**B**) Proportion of apoptotic (Apo 2.7 +) non-$CD38^{+}$/$CD45^{-}$ primary stromal cells after 72 hours of Tegavivint, panobinostat or the combination was validated. The expected drug combination responses were further calculated based on Bliss reference model using SynergyFinder. Deviations between observed and expected responses with positive and negative values denote synergy and antagonism respectively. (Due to the use of a single dose of panobinostat, synergy score calculation and interpretation has limitations).

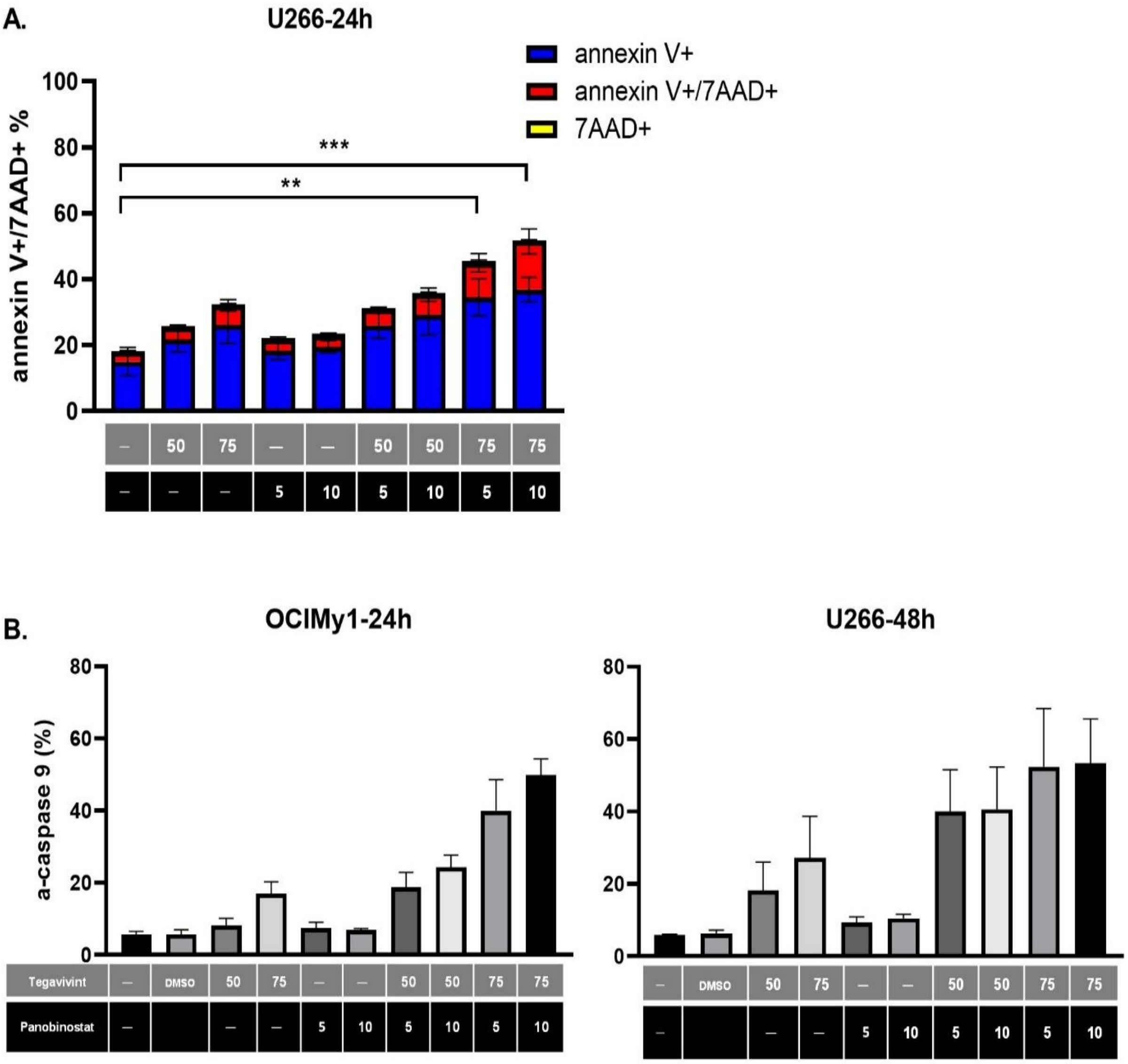

Figure S3. (**A**) Percentage of annexin V, annexin V/7AAD and 7AAD positive cells after vehicle alone (DMSO), Tegavivint (50 and 75 nmol/L), panobinostat (5 and 10 nmol/L) and combination treatment of U266 for 24 hours ($n = 3$, ± SE). (2-way ANOVA Dunett's multiple comparisons test with control row mean DMSO, ** $p = 0.0029$, *** $p = 0.0002$). (**B)** Percentage of active caspase-9 after treatment with Tegavivint, panobinostat or the combination for OCIMy1 and U266 at 24 and 48 hours respectively (raw data).

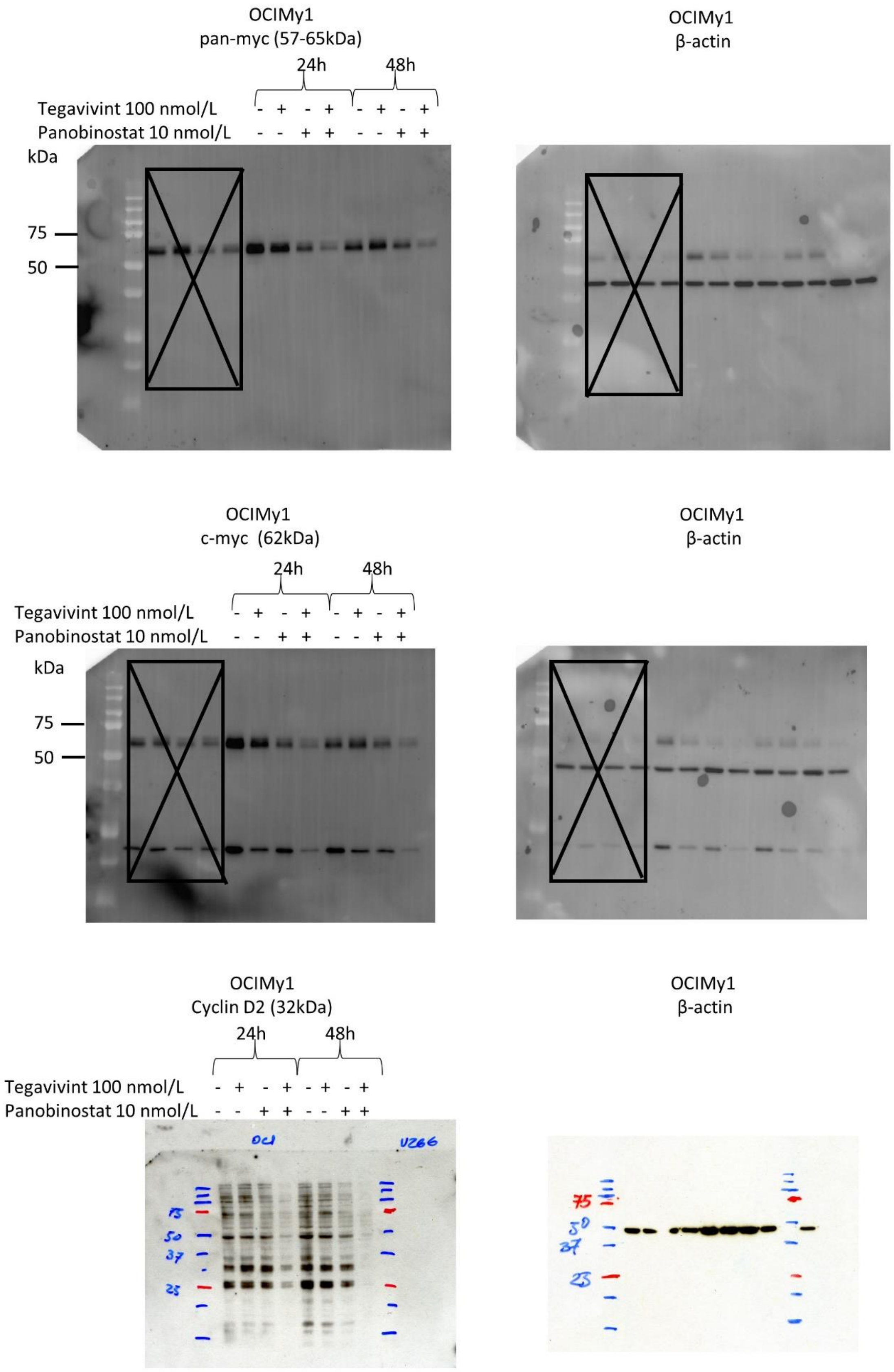

Figure S4. *Cont.*

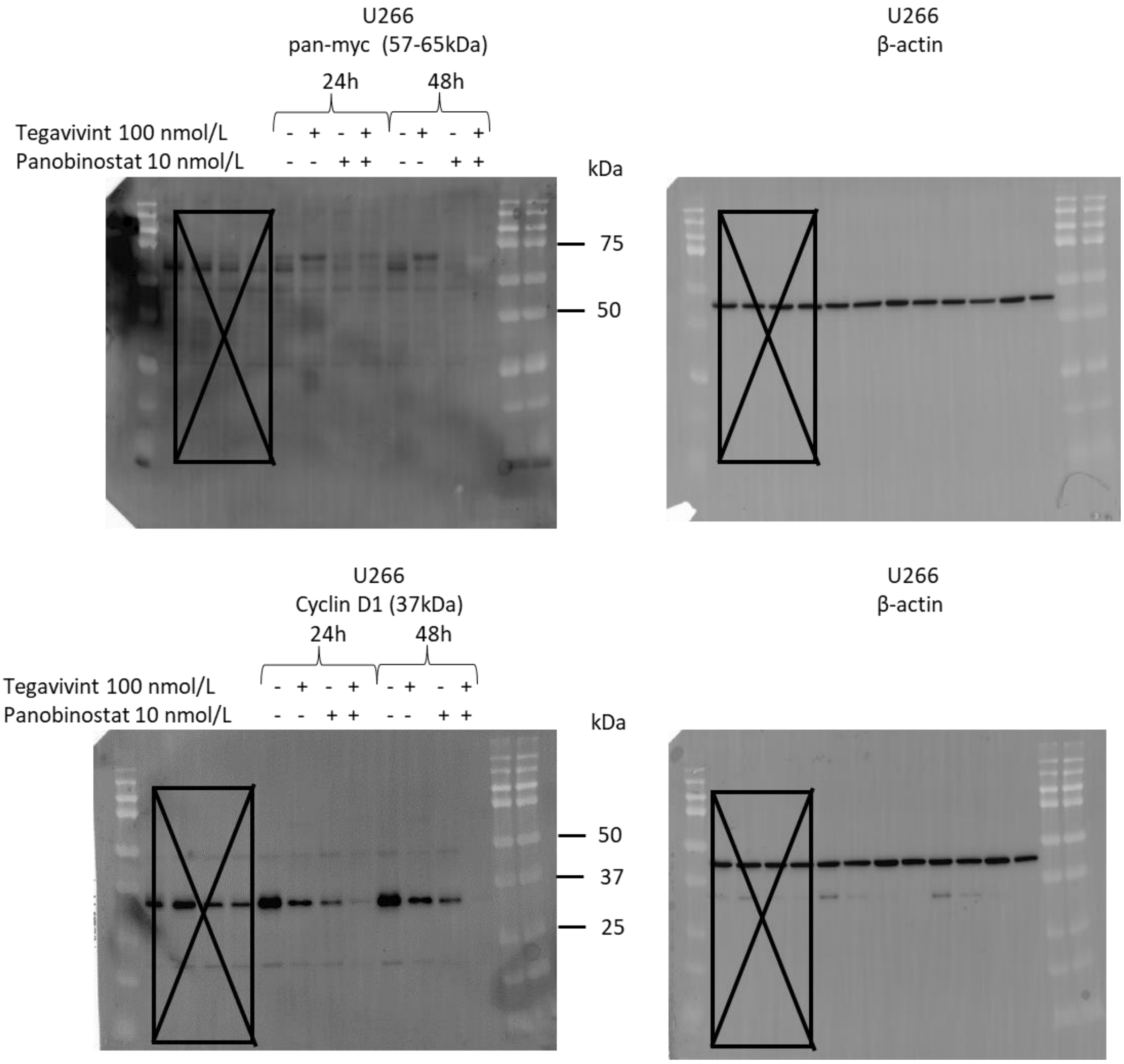

Figure S4. Composite images (chemiluminescent and colorimetric)/scanned images (cyclin D2) derived from immunoblotting of OCIMy1 and U266 whole-cell lysates for β-catenin down-stream targets Myc, Cyclin D1 and Cyclin D2 after 24 or 48 hours of treatment with Tegavivint, panobinostat or their combination. Loading control: β-actin.

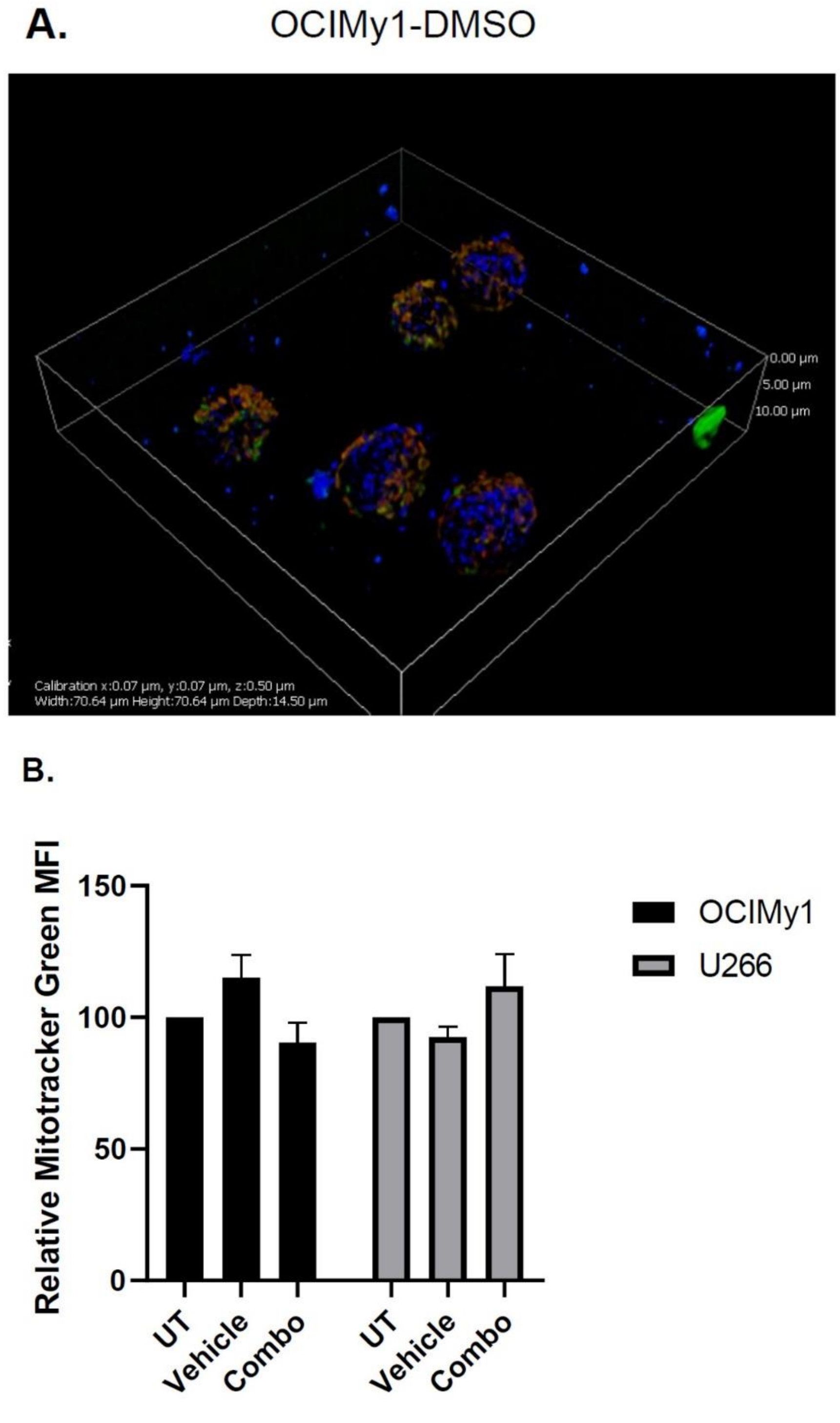

Figure S5. (**A**). Large view for scaling information of OCIMy1 replicate treated with DMSO for 20 hours and stained with TMRE (red for mitochondrial potential), Mito Tracker Green (green for mitochondria) and Hoechst 33342 (blue for nuclei) and evaluated with confocal microscopy. (**B**) OCIMy1 and U266 were treated with 100 nmol/L of Tegavivint in combination with 10 nmol/L of panobinostat or vehicle (DMSO) for 20 hours. Mitochondrial load was measured by Mitotracker Green straining and FC. MFI was measured and expressed as a relative % to the Mitotracker Green MFI of untreated (UT) cells (UT = 100%).

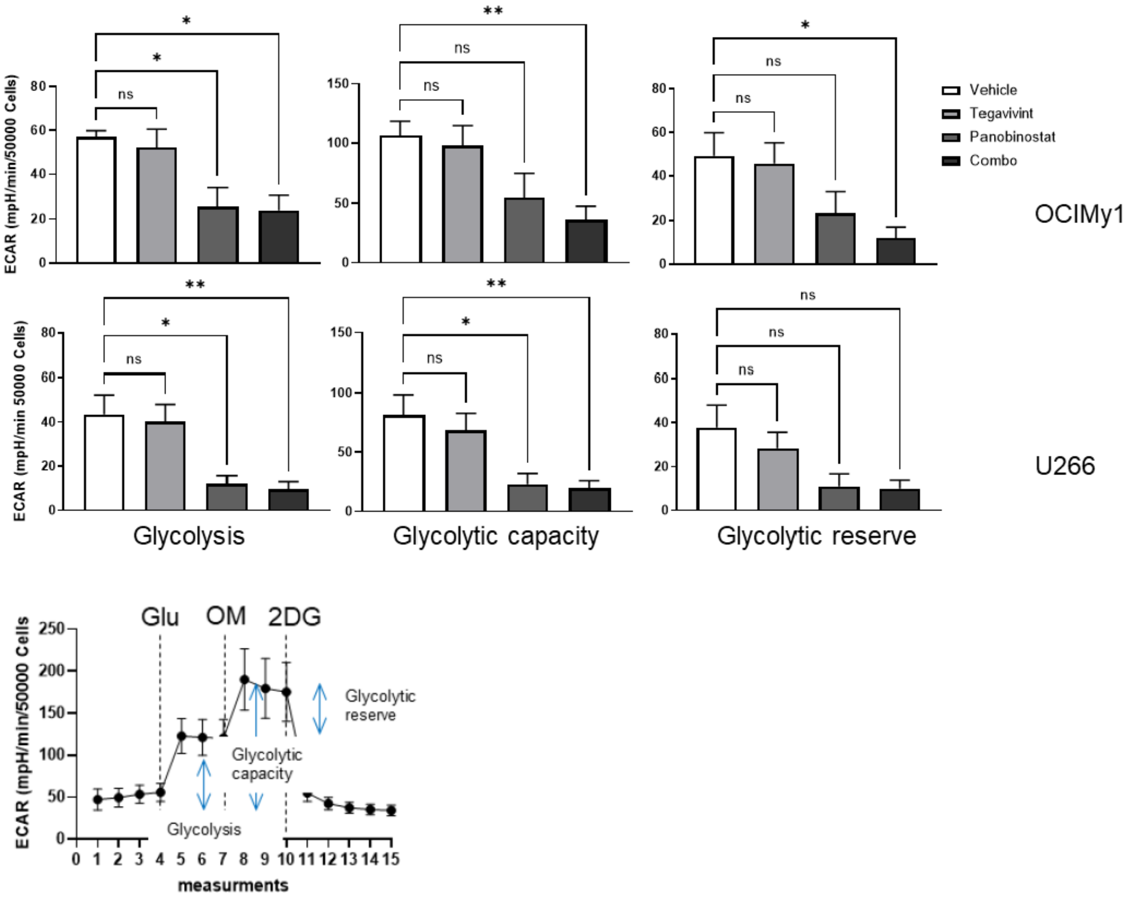

Figure S6. OCIMy1 and U266 were treated with vehicle, 100 nmol/L of Tegavivint, 10 nmol/L of panobinostat, or combination for 18 hours. Analysis of ECAR was performed using Seahorse XF analyser to assess glycolysis in the context of a glycolysis stress test. Three technical replicates were performed for each experimental variable (n = 3, ± SE). Results from ECAR analysis performed using Seahorse XF analyser were further analysed and basal glycolysis, glycolytic capacity and glycolytic reserve for OCIMy1 and U266 were calculated (n = 3, ± SE) (nonparametric Kruskal-Wallis multiple comparisons test * $p \leq 0.05$, ** $p \leq 0.01$).

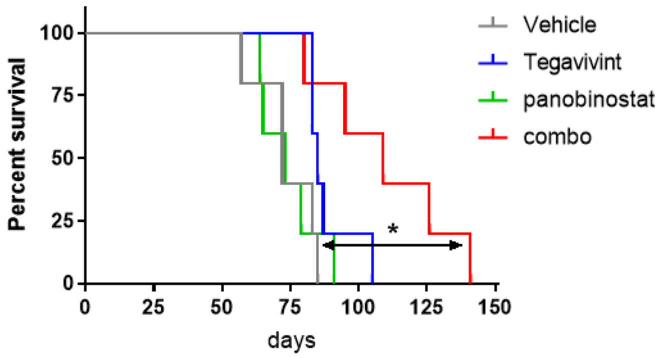

Figure S7. NSG human MM–bearing mice were treated with vehicle alone, 10 mg/kg of panobinostat, 30 mg/kg of Tegavivint or the combination of panobinostat and Tegavivint. Kaplan-Meier survival curves of the four cohorts are shown (* p= 0.01, Logrank test performed).

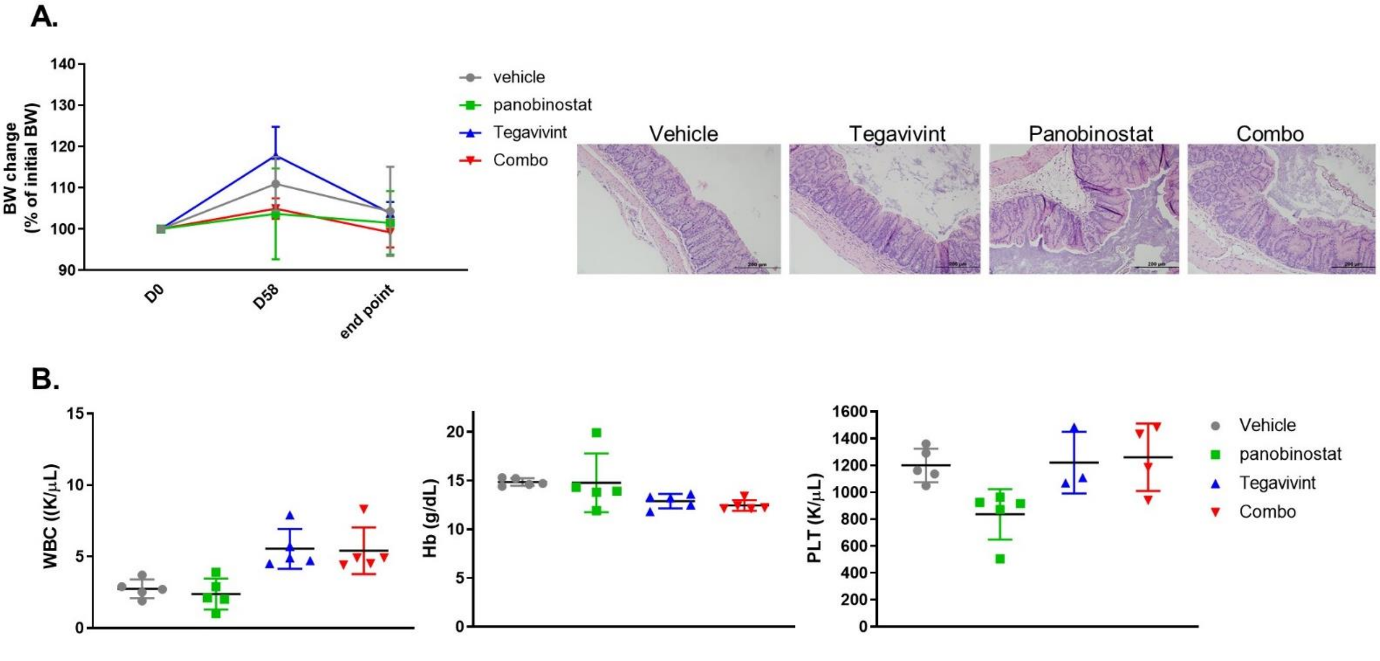

Figure S8. (**A**) Panobinostat alone, Tegavivint alone or combination treatment did not affect the body weight (BW) of the MM–bearing mice. 2-way ANOVA was used for statistical analysis, and no significant difference in bodyweight was found between the groups throughout the progress of the disease. IHC of colonic mucosa was normal in all treatment arms. (**B**) At completion of two treatment cycles (day 57) blood was collected and cell counts were performed. No significant differences in blood counts were found between the combination treatment and vehicle arms.

1. Supplementary Materials and Methods

1.1. Myeloma Cell Lines

The authors have confirmed that the cell lines are plasma cells by CD138, CD38, and CD45 by flow cytometry. All cells were cultured in a humidified incubator at 37°C with 5% CO_2 and used until 20th passage. All HMCLs were passaged 24 hours before the experimental setup to ensure high viability. Cell lines are screened every 2 months for mycoplasma contamination by VenorGeM Mycoplasma Detection Kit. The HMCLs are regularly authenticated by CellBank Australia by the use of short tandem repeat profiling, in line with the standard ANSI/ATCC ASN-0002-2011 and matched publicly available data.

1.2. Primary Samples Ex Vivo Treatment

BMMCs were isolated with Ficoll-Paque Plus (Amersham Biosciences Uppsala, Sweeden), washed in PBS, and red blood cells were lysed with NH_4Cl solution (8.29 g/L ammonium chloride, 0.037 g/L ethylene diamine tetra-acetic acid, and 1 g/L potassium bicarbonate). Cells were then washed with PBS, quantitated by hemocytometer, and subsequently cultured in RPMI1640 media supplemented with 10% heat-inactivated FBS and 2 mmol/L l-glutamine for 24 hours. The percentage of multiple myeloma cells was quantified by CD45 and CD38 staining by FACS. The next day, cells were plated at 2.5×10^5 cells/mL and treated with Tegavivint (250–1000 nmol/L) alone or in combination with panobinostat (10 nmol/L) for 48 hours. Drug-induced multiple myeloma–specific cell apoptosis was then compared with untreated controls by staining for CD45 FITC (BD Biosciences, Franklin Lakes, NJ, USA), CD38 PerCP-Cy5.5 (BD Biosciences, Franklin Lakes, NJ, USA), and Apo 2.7 PE (Immunotech Beckman Coulter, Marseille, France) followed by FACS analysis. SQ were calculated as the ratio of the cell death caused by the combination divided by the cell death induced by each drug alone at the same concentration.

1.3. Immunoblotting

OCIMy1 and U266 cells were treated for 24 and 48 hours, collected and lysed with RIPA buffer. 30 μg of protein were subjected to SDS-PAGE and transferred to polyvinylidene fluoride membranes (PVDF) (Merck Millipore, Burlington, Burlington, MA, United States). The PVDF membranes were then incubated with primary antibodies (pan-myc

[Abcam], c-myc [Cell Signaling], cyclin D1 [Santa Cruz], cyclin D2 [Cell Signaling]) at 4 °C overnight, followed by incubation with HRP-conjugated anti-rabbit or anti-mouse secondary antibodies (Dako, Melbourne, Australia) for 90 minutes at room temperature. Immunoreactive bands were visualised by ChemiDoc™ MP Imaging System (Bio-Rad, Hercules, CA, USA).

1.4. Flow Cytometry

The degree of cell death with different treatments was assessed at 48 hours by FACS with propidium iodine (PI) staining (62.5 ng/mL [Sigma-Aldrich, Merck Pty. Ltd., Bayswater, Australia]). The expected drug combination responses were calculated based on Bliss reference model using SynergyFinder. To study the proapoptotic effect of Tegavivint, panobinostat and their combination HMCLs were treated for 24 and 48 hours, and the cells were stained with FITC-Annexin V antibody (Molecular Probes by Life Technologies, Waltham, MA, USA) for 30 minutes in the dark at room temperature, washed, resuspended in Annexin buffer with 7AAD (Biolegend, San Diego, CA, USA), and acquired by FACS.

For active caspase 9 (a-caspase) experiment OCIMy1 and U266 were treated as before, for 24 and 48 hours, stained with a-caspase 9 (FITC-LEHD-FMK) according to the manufacturer's instructions (Abcam, Waltham, MA, USA) for 30 minutes in the incubator, washed, resuspended in FACS buffer (0.5% heat-inactivated FBS in PBS) and analysed immediately by FACS.

For TMRE and Mitotracker Green FM staining OCIMy1 and U266 were treated as before, for 20 hours, stained with TMRE (100 nmol/L) or Mitotracker Green FM (200 nmol/L) for 30 minutes in the incubator, washed, resuspended in FACS buffer (0.5% heat-inactivated FBS in PBS) and analysed immediately by FACS.

1.5. XF Glycolysis Stress Test

2×10^5 cells/mL of OCIMy1 and U266 were plated and treated with DMSO, Tegavivint, panobinostat or their combination. At 18 hours cells were counted, centrifuged and resuspended in the base medium containing 2 mM L-glutamine (Sigma-Aldrich) and seeded in a 96 well XF Cell Culture Microplate previously treated with Cell-Tak (Corning, Glendale, AZ, USA) according the manufacturer's instructions, at 5×10^4 cells per well. Drugs were added accordingly. The extracellular acidification rate was measured by XF^{e}96 extracellular flux analyser (Agilent Technologies, Santa Clara, CA, USA) with sequential injection of 10 mM glucose, 1 μM oligomycin A, and 50 mM 2-deoxy-glucose (Sigma-Aldrich).

1.6. Confocal Microscopy

Hoechst 33342 was excited at 405nm by a Coherent OBIS LX 405-100, 100 mW solid state laser and detected through dichroic cube 405/488/561/640 and an emission bandpass filter (450/50 Nikon, Japan). MitoTracker Green FM was excited at 488nm (Coherent OBIS 488-20 LS, 488 nm, 20 mW laser, Nikon, Tokyo, Japan) detected through an emission filter (525/50 Nikon, Tokyo, Japan), TMRE was excited at 561 nm (Coherent OBIS Coherent OBIS 561-20 LS, 561nm, 20mW, solid state laser) detected through an emission filter (595/25 Nikon, Japan). Images were sequentially acquired using VAAS detector (Virtual Adjustable Aperture System, Nikon, Tokyo, Japan) [1]. The detector uses two pinholes to collect emitted photons. A standard size and a second one with a diameter three times larger to capture additional photons without increasing the out of focus signal in a single scan. The signal is collected into independent channels, in focus and out of focus. Post-acquisition the data is added back and deconvolved using the extra collected information. The dXY image resolution was 0.21μm and Z interval of 0.5 μm. The images were deconvolved using 15 iterations of the TypeLandWeber algorithm using NIS Analysis Software (Nikon, Tokyo, Japan).

1.7. μCT analysis

All bones were supplied blind. The L5 vertebrae was identified using the last rib (the fifth vertebrae caudal from the vertebrae with the last rib junction) and sacral vertebrae (second last vertebrae before sacral hip vertebrae) as markers. The L5 bone was segmented from the vertebral column and analysed using optimal thresholding methodology available in the BoneJ plugin in ImageJ/Fiji. L5 vertebrae was segmented and the bone identified by thresholding with manual clearance of any artefacts or scatter. The volume, cortex and trabecular parameters were calculated following the thresholding step with the ImageJ-BoneJ plugin [2].

1.8. ELISA

Blood was collected before the injection of HMCL (healthy) and at the last week of cycle 2 in polypropylene tubes. After collection, blood was allowed to clot by leaving it undisturbed at room temperature for 20–30 min and then centrifuged at 2000× *g* for 10min at 4 °C. The resultant supernatant was immediately transferred into clean polypropylene

tubes and centrifuged again at 1000× *g* for 3 min at 4 °C. Clean supernatant was transferred in to new polypropylene tubes and stored in −80 °C until later analysed. For osteocalcin and C-telopeptide of type I collagen (CTX-1) measurements we used mouse Osteoclacin ELISA kit (sandwich ELISA) (LSBio, Seattle, WA, USA) and Mouse CTX-1 ELISA (Kamiya Biomedical Company) following the manufacturers' instructions while measurements were performed with FLUOstar OPTIMA plate reader (BMG Labtech, Ortenberg, Germany).

References

1. Okugawa, H. A new imaging method for confocal microscopy. *Proc. SPIE* **2008** ;*6860*, 1–7. https://doi.org/10.1117/12.774535.
2. Doube, M., Kłosowsk,i M.M., Arganda-Carreras, I., Cordelières, F.P., Dougherty, R.P., Jackson, J.S., Schmid, B., Hutchinson, J.R., Shefelbine, S.J. BoneJ: Free and extensible bone image analysis in ImageJ. *Bone* **2010**, *47*, 1076–1079.
